# Identity, Rurality, and Gender: A Phenomenological Exploration of Rural Nova Scotian Girls’ Physical Activity Experiences

**DOI:** 10.3390/children12040456

**Published:** 2025-04-01

**Authors:** Constance Tweedie, Laurene Rehman

**Affiliations:** School of Health and Human Performance, Dalhousie University, P.O. Box 15000, Halifax, NS B3H 4R2, Canada; ctweedie@dal.ca

**Keywords:** adolescent, girls, physical activity, rural, qualitative

## Abstract

Background/Objectives: Adolescent girls’ physical activity levels are well below the minimum recommended levels for health and wellbeing. Rural-dwelling adolescents may also experience decreased physical activity levels than their urban counterparts, placing rural adolescent girls at a greater disadvantage. The reasons for these low levels are multifactorial, including, but not limited to, age, geographical locations, and gendered stereotypes surrounding activities. Qualitative investigations into adolescent girls’ physical activity can provide deeper understandings on the key barriers and supports that specific populations experience and have been limited in the current literature. This study explores the physical activity experiences of adolescent girls living in rural Nova Scotia to provide deeper understandings of the needs of this population to inform physical activity policy and programming. Methods: Heideggerian hermeneutic phenomenology and feminist post-structuralism were used in a bricolage fashion to analyze six semi-structured interviews from adolescent girls between 11–13 years old living in rural eastern Nova Scotia. Results: Three major themes were identified from the interviews: (1) what physical activity looks like depends on your definition; (2) “What do you do when the boys take over the gym?”; and (3) “It’s really nice to have space…but there’s a lot less options out here”. The themes were not independent, but rather all were linked by the threads of gendered sociocultural expectations and hegemonic femininity. Conclusions: Distinct physical activity identities were exposed within the stories, shaped by parental and peer supports, personal ideals, sociocultural gender roles, and individual sense of agency surrounding physical activity engagement. Rural adolescent girls need both increased social and parental support to better navigate barriers of location and gender stereotypes that may be limiting their physical activity.

## 1. Introduction

Canadian children and youth are in a health crisis with 32% of school children considered to be overweight or obese [1], and few are meeting the minimum recommendations for healthy movement behaviors [2]. According to the Canadian community health survey, almost half of Canadian youth (54.3% of boys and 44.7% of girls aged 12–17 years) were meeting the minimum physical activity recommendations in 2018, a number that dropped to 39.5% for boys and 34.8% for girls during the COVID-19 pandemic in 2020. While these values did somewhat rebound by 2022 for boys (52.2%), they did not for girls (35%) [3]. While these low activity levels are concerning for all children, the prevalence for not meeting minimum standards of movement is even greater for girls [3,4].

Girls have historically engaged in less physical activity (PA) than boys [4,5,6] and begin dropping out of sport and PA at an earlier age than boys [7,8]. Much of this early decline in PA has been linked to gendered barriers [6,7], with girls choosing to engage in more sedentary socially accepted pastimes. This is particularly prominent during emergence into adolescence, when girls experience social changes and expectations related to gender norms that can be at odds with a physically active lifestyle [9,10,11]. In addition to age and gender, geographical location can also impact and influence girls’ ability and desires to engage in sufficient physical activity. Some authors have found that rural locations increase the barriers to physical activity experienced by adolescents [12,13]. The intersection of gender, age, and location therefore warrants deeper investigation into the possible ways in which rurality may be impacting the physical activity of adolescent girls.

As has been continuously observed in westernized countries, younger children engage in more PA than older children and youth [5,14,15,16,17]. Declines in PA have been observed from 47.6% in childhood to 24.4% in adolescents in Canada [5]. However, as children age, their likelihood of engagement in PA is associated with their level of PA in earlier childhood, with those who engaged in high levels of PA as young children becoming youth who engage in greater levels of PA than their peers [15], suggesting that maximizing childhood PA may reduce PA decline in adolescence. The observed decline in PA during adolescence is even more concerning for girls, with a noticeably higher decline in PA participation from childhood to adolescence in girls (35% to 14.1%) than boys (59.6% to 34.1%) [5,17,18,19] and increased reports of barriers to PA compared to boys [20]. Not only is there a drop-off in engagement, but the way in which children engage in PA as they age also changes from unstructured play-based activities to more structured (sport/dance class) activities [14] and decreasing time spent in outdoor play [17]. It is during this time, when children move from unstructured play to more structured PA activities, that there begins to be more stark contrasts between activities performed by girls and boys. With the transition from childhood to youth, researchers have reported higher participation of girls in socially influenced ‘feminine appropriate’ activities such as dance and gymnastics, whereas boys were more frequently engaging in ‘masculine appropriate’ activities such as sports [9,10,21,22]. This gendered difference may impact the choice of PA for all adolescents and may also result in choosing non-active social pastimes that align with their negotiation of expected gender ideals [9,21,22].

Although girls have been found to engage in PA for the same benefits as boys (e.g., social engagement, health, enhanced self-esteem, fun), [23,24,25,26] they also must contend with gendered stereotypes surrounding PA and concerns for personal safety [7,20,27,28]. Due to the continued existence of hegemonic femininity, girls navigate the social pressure to appear and act feminine, which is often contrary to many more vigorous and sport-based PAs [21,22,29,30,31,32,33]. Indeed, these gender differences in PA engagement have been observed in children as young as age five, with girls engaging in less vigorous PA and more lifestyle activities (dancing, bike riding) compared to boys who engage in more vigorous PA and more sport-type activities [14,23]. Girls who engage in typically ’masculine’ sports contend with sexuality stereotypes, that they are ’butch’, lesbian, or a tomboy. These stereotypes and social pressures often result in girls dropping out of typically perceived ’male’ sports [7,28,34]. It is therefore clear that adolescent girls must contend with sociocultural pressures to conform to expected gendered norms, which can have negative outcomes to their physical activity engagement [21,22,29,30]. These pressures, however, are not the only barriers that adolescent girls are facing with respect to physical activity engagement. Indeed, the local availability of various physical activity opportunities and the ability to access them can also impact girls’ activity levels. Recent research has begun to investigate the disparity in the urban–rural landscape and how geographical location can impact activity levels of youth.

The current literature from Canada and Europe investigating PA differences between rural and urban youth ranging from 8–14 years old has produced inconsistent findings. While some authors [12,13,20,35] conclude that rural youth are engaging in less PA and more screentime than their urban counterparts, others have found no difference [19] or have found rural youth engage in more PA than urban youth [36]. Canadian-specific data have also resulted in heterogenous findings, with Larouche et al. [37] finding no difference in PA levels between rural and urban youth ages 8–12 years while Rainham et al. [35] found rural youth between 12–16 years engaged in significantly less PA than urban youth. Some of this disparity in the literature may be due to the variable definitions of ‘rural’ between studies. While some studies use binary classifications of ‘urban’ and ‘rural’, others subdivide into three or more categories based on existing municipal boundaries or population levels [13,35], and yet others use a more nuanced classification system: considering population size, population density, and land use [12,20].

Alongside the level of PA experienced by rural youth, the supports and barriers to PA also appear to differ between rural and urban children and youth. In rural Canadian areas, barriers to PA reported by youth ages 8–14 are related to transportation and distance to places to engage in PA, as well as a lack of built environment such as sidewalks and bike lanes, whereas urban youth report traffic issues and the perception of crime [12,20]. Specific supports and barriers experienced by rural youth are dependent on the geographical, sociocultural, and historical context in which they live, and therefore rural experiences surrounding PA cannot always be transferred to other communities [38]. Likewise, PA policy and programming that was designed and/or implemented in urban or other rural locations may not meet the needs of all rural youth.

With the disparity in findings between urban and rural youth’s PA levels in the current literature, more focus needs to be given to the local sociocultural, and geographical, context when analyzing and interpreting findings. Acknowledging the heterogeneity of rural populations will help uncover the specific supports and barriers experienced by today’s rural youth and therefore will aid in tailoring policies and programming to better support these subgroups.

Rural eastern Nova Scotia in Canada was selected for this study due to the population’s poor PA levels, health outcomes [39,40,41], and high rural population: between one quarter to one half of Nova Scotia residents live in rural locations [42]. There is scant literature investigating youth PA in rural eastern Nova Scotia, with most NS-specific investigations centering around the Greater Halifax metro area (the largest city in the province). Due to the inconsistent findings on rural youth PA [12,19,35,37,43,44], the increased risk for girls to engage in less PA as they enter adolescence [5,18,45,46], and the need for greater understanding of location-specific supports and barriers [38], an in-depth investigation is needed to understand the lived experience of this underrepresented population. Therefore, the purpose of this research was to explore the lived experience of adolescent girls living in rural eastern Nova Scotia, Canada, to better inform local policy- and decision-makers with future PA programming.

## 2. Methods

### 2.1. Methodological Approach

The data used in this paper are a part of a larger study exploring the relationship between rural mothers’ and daughters’ physical activity experiences. Six rural mothers (mean age 41.3 years) and their adolescent daughters (mean age 12 years) participated in separate one-on-one interviews with the lead researcher. Within this paper the rural daughters’ lived experiences of their own physical activity are presented. The purpose of this paper was to explore the lived experiences of PA, specifically, how the barriers, supports, and attitudes surrounding PA affect engagement, how the gendered discourses surround girls and impacted their PA choices, and how living in a rural community influenced their PA.

This paper used Heideggerian hermeneutic phenomenology (HHP) [47,48] with an interpretative lens of feminist post-structuralism [49,50,51] to fully understand the participants’ experiences of physical activity, as it relates to, and interacts with, all parts of their lives. As Pelletier [52] explained, “Exploring physical activity participation using qualitative research methods can provide an in-depth exploration of the experiences and perceptions of participation in a specific social context, providing rich data to inform population and public health strategies” (p. 691). Performing methodological pluralism between these two epistemologies creates a more robust knowledge construction than either one alone as both view experiences and understandings through the lens of the specific time and place. HHP understands that the culture, historical time, and power dynamics at play can impact both the participants’ recounting of their experiences as well as the researchers’ understandings and interpretations of those experiences. Utilizing the principles of feminist post-structuralism outlined by Aston [50] as a guiding framework while engaging in the hermeneutic circle creates the opportunity to not only explore the meaning given to physical activity experiences, but also to uncover gender-based power relations that are imposed on the participants which may impact their PA experiences, which HHP traditionally does not address. Pluralism between HPP and feminist theory has been implemented similarly in existing literature [53,54]

This ability to understand experiences within the local sociocultural environment and in the current historical time will allow a deeper revelation of what current rural adolescent girls’ lived experiences are. Due to this, both methodologies also require the researchers to acknowledge their own situatedness, using methods of reflexivity to help bring forth unconscious biases, privilege, personal and political beliefs, and understandings that have been shaped by the researchers’ experiences, culture, and time. In so doing the researchers are better able to limit placing their own beliefs upon the experiences of the participant and can become more open to understanding and interpreting the lived experiences of others [55]. By using reflexivity throughout the entirety of the project, and using the hermeneutic circle, Heideggerian hermeneutic phenomenologists aim to expand their horizons of understandings by merging their own “Dasein” (ways of being in the world) with that of the participants [47]. The convergence of HPP and feminist post-structuralism in this study will allow a deep understanding of the lived experiences of adolescent girls living in rural Nova Scotia and will help to uncover how social, cultural, geographical, and gendered experiences impact their desires and ability to engage in physical activities.

### 2.2. Situating the Researcher

Author: I am a Caucasian, middle-class, mid-life, cis-gendered woman in a doctoral program. I am also a mother of three daughters, living in a rural Nova Scotian community. My experiences as a mother of an adolescent girl living in a similar geographical location to the participants have shaped my understandings of how social, cultural, historical, geographical, and gendered impacts have played a part in my own daughter’s PA experiences. Reflexivity on how I relate to my daughter’s lived experiences has allowed me to acknowledge my personal bias and situatedness, allowing greater acceptance and understating of various and opposing views from my own initial and personal experiences.

### 2.3. Situating the Participants

All the participants interviewed were Caucasian middle-class girls, mean age of 12 years, living in rural eastern Nova Scotia. This region has a median income of CAD 62,400 and a population density of 13.8 people per square km [56]. Detailed demographics of the participants can be found in Table A1.

### 2.4. Method

This study was approved by Dalhousie University’s Institutional Research Ethics Board REB #2023-6867. Adult participants (mothers) were recruited via a social media flyer, inviting the mother and their daughter to participate in one-on-one online interviews with the lead researcher. Separate informed consent forms were signed by both the mothers and daughters, and verbal assent was given by the daughters at the beginning of their interviews to endure voluntary participation. Six semi-structured interviews were conducted between 27 January–2 May 2024, using online conferencing software, and lasted between 20–45 min. Sample size was selected based on previous similar studies [57]. The theoretical underpinnings of Heideggerian hermeneutic phenomenology, which proposes that “given that an individual person can generate hundreds or thousands of concepts, large samples are not necessarily needed to generate rich data sets” [58] (p. 1374), were adhered to. The interview questions focused on the lived experiences, barriers, supports, and attitudes surrounding PA for adolescent girls living in rural Nova Scotia. As this paper is a subsection of a larger study, only interviews with daughters are reported in this manuscript. Examples of interview questions can be found in Table A2. Ultimately, the interview questions explored how being an adolescent girl and living in a rural location impacted their physical activity desires and abilities. In reporting on the findings, I use pseudonyms to ensure anonymity. Due to the small geographical location samples, any other potentially identifying information has been removed from the quotations used in this report to ensure anonymity.

### 2.5. Data Analysis

Interviews were recorded, automatically transcribed via a voice-to-text application, and then manually verified for accuracy by the lead researcher. HHP analysis was carried out using a six-stage framework as outlined by Ajjawi and Higgs [59]: (1) immersion, (2) understanding, (3) abstraction, (4) synthesis and theme development, (5) illumination and illustration of phenomena, and (6) integration and critique. During the phenomenological analysis, the principles of feminist post-structuralism outlined by Aston [50] were used as a guiding framework: (1) power as relational, (2) binary opposites, (3) regulated communications, (4) feminist theory, (5) discourse analysis, (6) language and meaning, (7) beliefs, values, and practices, and (8) subjectivity and agency.

Stage one, immersion, was achieved by reading and re-reading the transcripts and making notes and annotations on each transcript. Stage two, understanding, involved the construction of participant codes, using their own words. Stage three, abstraction, involved creating researcher codes and grouping these codes into sub-themes. Common participant codes for each participant were grouped based on research sub-questions, then the source material for each code was reviewed to better understand and interpret the essence of the sub-theme. Stage four, synthesis and theme development was performed by reviewing the researcher sub-themes and annotations and comparing sub-question coding across participants. Here, immersion into the hermeneutic circle occurred, as the understanding of the parts (codes and sub-themes) involved the researcher reviewing transcripts to understand/interpret how the whole could be seen differently by the understanding of the parts. It was through the process of engagement in the hermeneutic circle that final theme development occurred. Stage five, illumination and illustration of phenomena was undertaken as the researcher returned to the literature to place the findings in context. Finally, stage six of integration and critique was completed in the production of this manuscript which will allow critique and discussion by others. Examples of how raw data were developed into final themes are present in Table A4.

Throughout each stage of analysis, the researcher engaged in reflexive journaling using reflective questions posed by Valandra [60] and Duffy [55] as a guide. See Table A3 for examples of reflective questions. In addition to reflective journaling, I engaged in frequent discussions with my supervisor on the developing themes, to bring forth and move beyond my situated biases.

### 2.6. Data Presentation: Creation of Vignettes

Composite vignettes were created using participants’ own words to exemplify each emergent theme. The use of composite vignettes (crafted vignettes from multiple participants’ interviews that speak on the same theme) have been used by other phenomenological researchers to illustrate the interpretative understanding of multiple lived experiences [33,59,61]. The use of composite vignettes as a means of data presentation has been argued to align well with the philosophical underpinnings of hermeneutic phenomenological research, allowing a portrayal of the researcher’s interpretation while using participants’ own words and stories to express the underlying experience of each theme [48,62]. Following coding for themes (stage four above), experiences and stories from multiple participants were crafted into cohesive vignettes by the first author using participants’ own words, to effectively illuminate and illustrate each major theme (stages four and five), as if from one viewpoint.

## 3. Results

Three major themes emerged from the hermeneutic analysis: (1) what PA looks like depends on your definition; (2) “What do you do when the boys take over the gym?”; and (3) “It’s really nice to have space…but there’s a lot less options out here”. These themes were not distinct from one another, as the participants’ PA identity (outlined below) appeared to influence their perceptions and reasons for participating.

### 3.1. What PA Looks Like Depends on Your Definition

The theme “what PA looks like depends on your definition” is an interconnected picture of how each participant’s definition of PA and PA identity impacted their experiences surrounding PA participation. The participants in this study appeared to have two distinct interpretations and internalizations around what PA entailed and how/why they engaged in physical activity. The participants who defined PA as ‘sport’ (Janet, Jenny, and Ginny) appeared to have a “sports girl” identity and expressed different motivations, beliefs, supports, and barriers surrounding PA than the participants who defined PA as any type of physical movement (Sally, Lillian, and Sharon), which was often in nature (hereafter called the “nature girls”).

The “sports girls” associated PA with sports, participated in competitive sports (soccer, baseball, combat sports) and did not view lower-intensity activities (such as gardening and casual walking) as being physical activity; rather, they defined these activities as simply non-sedentary. The “nature girls” had a much broader view of what constituted PA and tended to make a connection between the outdoors/nature and physical activity, often expressing that ‘being outside’ was to be physically active. In contrast, the ‘sports girls’ expressed much less of a link between the outdoors and PA even though they engaged in outdoor activities. All the participants noted that engaging in PA was important, and that it provides both physical and mental health benefits, although few were able to give clear definitions of what ‘being healthy’ meant to them. The following vignettes outline the two distinct PA identities expressed by the participants.

Sports girls: vignette crafted from Janet’s and Jenny’s stories:

*PA is like sports and like running and just stuff in that kind of area, I guess. Things like walking and gardening might be a bit, but not really, not how I would define it. I do soccer, dance, and [martial arts]. I do a lot of sports, but I feel like I could be doing more, but that’s just my thought, because I feel like I want to be a more like an active and like fast kind of girl. I might like to do CrossFit and track or something, something that involves, like lots of like more physical activity, because it’s like meant for kind of helping you be better at running and like lifting stuff. I feel like it just might help me like, later on. And also it’s like good for your health to keep up and do some exercise every now and then, I feel like it just makes me happy. You’re doing exercise, like just getting up and doing stuff, and also people told me it’s good for your health, which also makes it even better because it makes me feel like I’m doing something right.*

Nature girls: vignette crafted from Lillian’s and Sally’s stories:

*When I think about PA my brain automatically goes to like doing things physically with your body like, just going outside to get fresh air or to get exercise or something. I think I’m pretty active, but like, differently at different times. I do laze around on the couch a lot, but I do play and stuff, and I like to go on hikes or do an exercise video or something. I guess I’m just not very consistent with what I’m doing, like sometimes we’ll go for like a swim one weekend or the next weekend we’ll go for a hike or something. I know PA is important, because I’m young and should be getting a lot of exercise to stay healthy, and like mentally healthy too because, like going out for fresh air keeps you mentally healthy. You know, usually if I’m feeling a little cranky, I get outside and after a little while, I start to feel a bit better, it feels pretty nice to get out in the fresh air after sitting on the couch all day.*

The majority of the participants felt they were getting sufficient physical activity and a similar amount to their peers (except for Janet who thought she was more active). This was a surprising outcome, as their self-reported PA levels ranged from participating in three structured activities (up to five days/week) to participating in no structured activities. For all the participants, the school provided consistent PA opportunities, with physical education (PE) two to three days per week as well as outdoor education and yoga. Intramural and active play during the school hours (at lunch and recess) was not identified as a consistent means of obtaining physical activities, with only one participant (Janet) participating in intramurals. As will be discussed in the next theme, gendered production was limiting the participants’ desire to participate.

Vignette crafted from Sally’s and Sharon’s stories:

*We have physical education two or three times a cycle, it’s a lot of sports: basketball, soccer, soccer baseball, and we did do archery. I don’t like all sports, but I like most of them. We also have this thing called outdoor education once a cycle, which is just all outdoor PA and stuff, last week we went snowshoeing because there’s like trails behind our school and sometimes, we go for hikes, or we build shelters. And we have a yoga class once a cycle, which is kind of active, we do some stuff like downward dog and the cobra and child’s pose, but then it’s also some like meditating, self like reflections, stuff. That’s about it at school, not much activity happens at recess or lunch, we’ll just walk around or talk with other friends and kinda just hang out, there are some boys who play football, umm, that’s kind of it though.*

The experience of trying new activities varied with the participants’ PA identity. Nature girls’ attitude around trying new activities centered around needing to already be good at it and to have friends with them for social support. They expressed significant lack of confidence, feeling awkward, and a fear of failure when engaging in new physical activities. The need for friends to be co-participating for the nature girls was a strong support to reduce the feelings of judgment. These participants found they needed more extrinsic encouragement to engage in PA, and found support from friends, family, nature, and, somewhat surprisingly, from social media.

Vignette crafted from Sally’s and Lillian’s stories:

*I would only want to join a new activity if I’m good at it, if I enjoy it, and if it’s easy. Also definitely having friends in it, people I know. If I do it alone and I’m not good at the sport first, then I’m gonna be very embarrassed and awkward. Like I wouldn’t want to try hockey, because I’m not a very fast skater, so it’ll just kind of feel like, slow and not fun. But I started dance this year with my friends and it’s better with someone else because then it’s just more fun*.

Vignette crafted from Lillian’s and Sharon’s stories:

*Sometimes I’ll watch a movie or see a TikTok or something that is just people outside having fun and doing stuff. And then I’m just like “I wanna do that”. So, I go outside and do that. But sometimes I need like encouragement to do it, like sometimes I’ll be able to get myself the encouragement, but I also like I don’t wanna go outside alone and I don’t really have another person to hang out with and do stuff with. So, I normally just try to like get my parents to come.*

Sports girls’ attitudes around trying new physical activities were much less focused on having friends to initiate participation but rather doing the sport helped to make friends. They also had high internal motivation to engage, even when they did not ‘feel like it’ (*excerpt from Janet’s story*), and found engagement in competition to be a strong motivator for sport participation. The sports girls had high logistical support from their families, as they needed to travel into town to participate. Unlike the nature girls, none of these girls mentioned using social media (TikTok, YouTube) to engage in PA, but rather as a sedentary activity.

Vignette crafted from Janet’s, Ginny’s, and Jenny’s stories:

*Sometimes I join some things because my friends are in it, but then after a while I realize I’m still doing it not because my friends are in it, but because I like it. But, like, if I want to try a sport, I don’t need my friends to join for me to join. I do sports because they’re really fun and it also gives me that exercise time, so I just really like it. I like competitive things a lot, I like the pressure. It’s like when you’re going against a really hard team and you know they’re good. It’s like it feels really good to, like, have a pep talk with your team and then like, getting put in the field and you’re just ready and like until the whistle blows, it’s really the anticipation. I guess I really love just playing it. Which is why even when I’m really exhausted, sometimes I don’t really want to go to practice but I always go anyway and I’m happy that I go in the end because I know I’m always gonna be happy that I did go*.

Only two participants associated PA with ‘play’ and were the two youngest participants (Jenny and Sharon). Both girls recognized that many of their peers no longer engage in ‘play’ the way they do. The adolescent transition away from play was highlighted by the oldest participant (Lillian), who emphasized how she does not want to engage in PA with slightly younger kids as she feels she is ‘older’ and is actively seeking out activities to engage in with people her own age, moving away from the play mentality. The two opposing experiences, ‘play-based’ and ‘away from play’, are highlighted in the following two vignettes.

Vignette crafted from Jenny’s and Sharon’s stories:

*I think my PA experience is different from a lot of the girls my age. A lot of my PA is like going outside and playing in the snow or in the grass, or playing with our dogs, maybe playing with some games with my mom. Everyone other than like 2–3 friends I’ve seen, they usually don’t really play, and it’s just like they wanna be like 16- or 18-year-olds. It’s like they’re wearing makeup, they’re wearing dresses, they put their hair up in a ponytail and they go “Hey guys this is my skin care routine”. So, most of the people that I see play is just like me and a couple of my friends. Sometimes we’ll go sledding or play baseball or go exploring around, sometimes we’ll play with the dogs. When it’s snowing, I’ll put my snow pants on and I’ll jump in the snow. And other kids in my school just stay inside and don’t really go outside. They just wanna sit there, they just wanna go to the bathroom and talk, but honestly, I don’t really care about it because I just like running around, so I just kind of do that*.

Extract from Lillian’s story:

*I really enjoyed [martial arts] and I stayed in that for a while. Maybe like three years but then I did stop a little over two months ago. It was fun at first, but then it started turning into a kid’s class. When I first started it, it was women, like teenagers and stuff but then more people started knowing about it, and a lot more like grade sixes or grade fives start joining in and I don’t know, umm, I don’t really like kids a little younger than me. Since I’m 13 going on 14, I kinda want people my own age or older. So, I’m looking for something maybe like 13 to 16 age group.*

Rural girls’ experiences around PA cannot be grouped into one singular understanding, but rather each girl’s PA experiences were impacted by their PA identity, geographical location, and age. Those who adopted a sports-girl identity defined PA as ‘sports’, engaged in more structured PA, had high internal motivation for PA and logistical support from their families to achieve their PA desires. The participants who adopted a nature-girl identity defined PA broadly as any bodily movements, had a strong connection between PA and nature, engaged in less structured activities, and needed high peer support for PA engagement. Amongst the oldest four participants (Lillian, Janet, Ginny, and Sally) there was a shift away from play-based activities whereas the younger participants (Jenny and Sharon) still engaged in unstructured play. While school PA classes (PE, yoga, outdoor education) offered participants consistent opportunities for PA, existing opportunities for free-time PA (i.e., intramurals) at school were not engaged in by more than Janet. This lack of participation in school free-time PA appears to be impacted by the gendered monopoly of the school yard/gyms, as will be discussed in the following theme.

### 3.2. “What Do You Do When the Boys Take Over the Gym?”

When asked how being a girl impacted their choices and engagement in physical activity, all but Ginny expressed that being a girl did not impact their physical activity. As Sharon voiced, they could do “whatever they want”. However, when describing experiences surrounding physical activity, most of the participants described incidents where boys were creating an unwelcoming atmosphere for girls to engage in sports/physical activities at school, which diminished their desire to participate in certain activities. The participants acknowledged the gendered control of athletic spaces that was occurring around PA at school but accepted this as the norm, lacking agency to intervene. In speaking about community sport, many of the participants referenced how most sports had gender segregation, and they viewed this as a benefit, as it allowed them to play without feeling belittled. Jenny’s experience participating in an all-girls team (excerpt below), with female coaches, highlights the potential benefits. The following three vignettes exemplify the sub-themes of “I can play whatever I want”, “I don’t want to play with a bunch of dudes”, and “the girls don’t play because the boys take over the gym”.

“I can play whatever I want”: Vignette crafted from Janet’s, Lillian’s, and Sharon’s stories:

*They’ve kind of got boys and girls teams for everything or they have teams that involve both, both boys and girls. Because girls can do boy stuff and boys can do girl stuff and it’s just if a girl wants to play basketball, she can play basketball. If a boy wants to play dress up, he can play dress up. It’s fine. I don’t think any sports should be ruled by boys or girls. I think that anyone can play if they want to. Oh, but like most sports, like people think that boys are better at it. So, it’s annoying hearing like, that’s like how everyone thinks, but it doesn’t matter. Usually I just like, don’t care. It doesn’t affect me. The only thing that would probably worry me is if there were guys watching me and coming in front of me because they think they know sports a lot better for some reason. But normally when you do sports, they divide girls and boys, so it’s kind of nice because then you have a lot of like girls doing a sport with me instead of like me and a bunch of dudes. Like one time I signed up to play basketball at lunchtime, and it was like, this is so awkward because it’s just one girl and it’s me and, I thought there was gonna be more girls there. I was like ‘God, please help me’ and like “What if I mess it up? What if the boys are like aggressive”, so it was a little like uncomfortable because the boys wouldn’t pass me the ball, because I’m a girl.*

“I don’t want to play with a bunch of dudes”: Extract from Jenny’s story:

*Last year, I was on a boy and girls team for [sport], but it wasn’t really that fun. Because there were only three girls that signed up, and they made three teams that would like to play each other you know? But then all the girls were put on different teams and the boys wouldn’t really talk to us or anything. They just kind of like play and talk to each other, you know. It was kind of boring and it wasn’t much fun either. But then this year I joined an all-girls team. At first, I didn’t know anybody, but the girls started to talk a lot, then I started becoming friends with them too. Now I’m gonna do that once again this summer because I have a lot of friends on that team.*

“The girls don’t play because the boys take over the gym”: Vignette crafted from Sharon’s and Lillian’s stories:

*Most of the time in my school the girls aren’t really doing much because they’re all just like walking around and the boys are like most of the time the only ones who like active playing. I know there’s a lot of girls in my age group that hate sports, and I wouldn’t say it’s because of the stereotype boys are stronger than girls, ’cause there are a lot of women stronger than guys. I do have a few friends who do soccer with me, but that’s really it. And still, they’re a little nervous to do it in front of the guys because [the guys] do like hockey, baseball, soccer, all kinds of stuff like that. And they just do a lot more sports and PA than we do, a lot of the girls in my class prefer reading and things like that. There are a lot of sporty guys in my class and they kind of take over the entire gym and then it’s kind of awkward for a lot of the girls. Like sometimes it’s just kind of embarrassing if you miss a ball or something ’cause since they’re all really athletic and really good at sports. They kind of, like, talk about you behind your back or maybe make fun of you. I know they do like to do that a lot and it’s quite annoying, but I don’t know, what can you do. I mean, I find I’m pretty good at sports, I think, but I don’t know. I haven’t heard them say anything to me, but I know they have said it to a few of the girls in my class and I feel bad, but I don’t know*.

This theme highlights a situation of conflicting cognitive belief vs. lived experience relating to gendered PA participation by adolescent girls. On the one hand the participants believe that they can play whatever they want to, that being a girl does not (or should not) limit their opportunities to play. On the other hand, they have experienced feeling unwelcome and bullied in PA spaces that are dominated by boys. While the participants refute that ‘boys are better at sports than girls’, they have not found an avenue to express their agency in defending their right to occupy these PA spaces.

### 3.3. “It’s Really Nice to Have Space…but There’s a Lot Less Options out Here”

Most of the girls found their rural location either neutral (Janet and Ginny) or beneficial (Jenny, Lillian, and Sharon) to their PA participation. For girls who had a deep connection between PA and the outdoors, their rural locations appeared to offer them greater opportunities to engage with nature. Only Sally expressed a fear of wild animals in the woods as a barrier, while Janet, Sally, and Ginny noted rural road safety to impact their participation.

Vignette crafted from Lillian’s and Jenny’s stories:

*It’s really nice to have space where we don’t have a lot of neighbors. We only have like four neighbors, so and we have a lot of yard space and a lot of places to go that aren’t really like restricted and stuff. We’re surrounded by, like, pretty big hills and the woods. We’ll go for hikes in our backyard, like yesterday we went for a walk down the road and found this cool trail and went in the woods and stuff. I really like walking through the woods, it’s very peaceful.*

Excerpt from Sally’s story:

*When I’m walking, I’ll just go like down the driveway and back. ’Cause I don’t want to go into the back woods, because there’s bears and coyotes*.

Vignette crafted from Sally’s and Janet’s stories:

*I don’t go on the road because it’s a pretty dangerous and fast road and I wouldn’t feel safe because there’s like lots of turns and like a car just pops up, like there’s lots of turns. That could just be like they just don’t see you until the very last minute. If I want to go for a long walk, sometimes we’ll drive down the highway and then there’s like a certain way you can take to go to the dirt road, which is like safer to go for walks on.*

For participants who lived farther from a town center (30–45 min, Sharon and Sally), there were increasingly fewer opportunities and limited sports teams (except hockey and soccer, which were noted as the major sports in town). This lack of opportunities for both unstructured PA and sport engagement was a major barrier and coincided with barriers surrounding seasonality and access to facilities. Those participants living closer to town (10–15 min, Janet, Jenny, Ginny, and Lillian) experienced fewer barriers related to transportation and expressed that their parents would drive them wherever they needed to go. These two different rural experiences are highlighted in the following two vignettes.

Vignette crafted from Sally’s and Sharon’s stories:

*Well, we live pretty far away from a lot of stuff, and right now our town pool is closed. So, if we wanna go swimming, we have to go all the way to the next town. And the town skating is also really short, so if we want a longer skate, we have to go farther away. There’s also less options in the winter here, ’cause in the summer we’ll go to the lake, which is only a couple minutes away, and at the Community Center, there’s also pickleball and you can go for bike rides. But there’s a lot less options in the winter around here. Unless you play hockey, everyone’s really interested in hockey, so that’s kind of it: hockey or soccer. That’s kind of mostly what you can do around here. Like, a while back I wanted to join gymnastics, but they only had it optional like far far away, so I couldn’t do it. And now I kinda wanna do basketball, but there is no basketball for me to go in at my school, so yeah.*

Vignette crafted from Lillian’s, Janet’s, and Ginny’s stories:

*My [martial arts class] is on the other side of town, we’re on one side, it was on the other side. So we have to drive all across town. Because we live so far, you have to really time it right to make sure you’re not too late, like when the roads are icy, you have to leave earlier because there is more ground to cover. But that’s the only issue I have getting to practice. My mom or dad always drive me to whatever is going on*.

The themes from this study highlight the diverse experiences that are had by rural Nova Scotian adolescent girls. They encounter both barriers and support in their PA endeavors, which vary depending on their PA identity, their experience of gendered barriers to physical activity, and living within a rural setting. Participants who held a sports-girl identity engaged in more frequent structured activities, had higher internal motivation for participating, and had higher PA rental logistical support. Participants who adopted a “nature girl” identity engaged in less structured and more sporadic physical activity. They expressed the need for more peer support and external motivation for participation and found social media to be a beneficial tool for motivating physical activity. The greater level of rurality was associated with greater sense of transportation barriers; however, rural participants also found support in their connection to nature and engaging in unstructured activities outside. All participants held strong beliefs that their gender did not limit what they could do, yet they experienced gendered barriers to PA within schools due to the perceived domination of athletic spaces by boys.

## 4. Discussion

In this paper, we explored the lived experience of rural Nova Scotian adolescent girls and their participation in PA, using HPP with a feminist post-structuralist viewpoint. The major themes that emerged from this exploration acknowledged the heterogenous experiences of rural adolescent girls, that varied depending on their beliefs, identities, age, and rurality. It also highlighted how the shared experience of gendered control PA spaces impacted their ability, desires and motivations to engage in physical activities.

### 4.1. Impacts of Identity and Rural Living on Physical Activity

One of the overarching themes that emerged from this exploration was how the participants adoption of either a “sports girl” or “nature girl” identity appeared to impact both their level and modality of PA engagement, as well as their perceived barriers and supports. Identity formation is “a complex, fluid phenomenon that is constitutive of and co-created through communication with others” [63]. During adolescence, girls are at a turning point in their athletic identity formation, navigating and internalizing sometimes conflicting messaging about their own perceived athletic abilities, parental and peer messaging regarding their abilities, and social messaging regarding athleticism and gender roles [64,65,66]. There is supporting evidence that during adolescence, girls are heavily impacted by social discourse surrounding gender roles, social interactions, and responsibilities, which may conflict with their desires to engage in PA [63]. Those who experience negative discourse around their athletic abilities, who have less parental support for athletic participation, and have fewer peers who support an athletic identity are less likely to adopt a positive athletic identity as they move through adolescence [63]. This athletic or “sports girl” identity was seen to impact the PA level and choices of the participants in this study. Those who adopted a “sports girl” identity participated in more structured activities, had greater self-motivation to engage, enjoyed competition, and had high parental logistical support, whereas the participants who did not ascribe to a sports-girl identity cited greater transportation and logistical barriers, had a broader definition of what PA meant, had a stronger reliance on peer support, and noted a greater association between PA and nature. These findings mirror those of Zuest [67] who found rural girls living in the Northwest United States had one of two PA patterns: *casual movers* and *sporty girls.* While the goal of this explorative study was not to ascertain the components of identify formation, it appears that parental support and transportation may have played a role in the adoption (or lack thereof) of a “sports girl” identity. As can be seen in the previous vignettes, a significant barrier reported by ‘nature girls’ was transportation. This did not represent a significant barrier to the ‘sports girls’, as was clearly stated by one participant: “My mom or dad always drive me to whatever is going on”. Not only does this highlight the heterogeneity in rural girls’ experiences, but it also exemplifies the need for parental logistical support to achieve their desired PA participation levels. This association between high PA engagement and high parental logistical support has been noted by others [68,69,70,71]. While the ‘nature girls’ did report greater transportation barriers than the ‘sports girls’, it should be noted that two of the ‘nature girls’ also lived in the areas of highest rurality. Both participants also mentioned that they would like to participate in various sports, but the lack of opportunity and transportation requirements were seen as prohibitive. This increased rurality of these participants exacerbated both the transportation and access to opportunity barriers experienced for rural youth [12,44]. The intersection between parental support and transportation requirements of those living in the most rural areas may be impacting not only rural girls’ PA levels, but also their PA identity. Unfortunately, deciphering whether the ‘sports girls’ developed the sporting identity due to their parents’ high support for sport participation, or if their parents’ logistical support was an outcome of their children’s strong desire to participate in sport, is beyond the scope of this paper.

While rural living impacted and increased transportation barriers for Sharon and Sally, rural living was not always seen as a negative. In line with other research, the participants in this study found their rural location provided both supports and a barrier to PA [13,43,44,67]. The participants in this study found that rural living afforded them increased access to nature, allowed for more unstructured PA, and was commonly associated as being supportive of their mental health.

### 4.2. Nature Experiences and Rural Living

The association between PA and nature was most prominently expressed by the “nature girls”, two of whom lived in the areas of highest rurality. Their experience of PA was directly connected to ‘being outside’, highlighting the importance nature had on their experience of health and physical activity. A similar impact of nature on girls’ health and wellness has been highlighted in the recent literature, with a positive connection found between nature and mental health [10], as well as between nature, PA, and mental health [29]. While the “nature girls” had a more direct association between PA and nature, the “sports girls” also commented on the mental health benefits of being outside. While living in rural settings increased the participants’ ability to access nature, they did not venture far independently; rather, their nature experiences were limited to their immediate surroundings, or where their parents took them on family outings (i.e., a hiking trail, the beach, etc.). Exploring farther into the woods or walking along the road shoulder (as there was no sidewalk access for any of the participants) was seen as dangerous and has been a commonly reported barrier to rural youth PA [12,29]. While the girls in this study did not engage in independent outdoor exploration (which may have been due to a variety of reasons, including age and sense of safety), this may be an area for future programming, as there is a growing interest in nature exploration and connection to nature by female youth [29]. The ability for adolescent girls to engage in more nature is something that will require navigation though socially ascribed gender roles as well as parental and peer support, as historically outdoor focused activities have been male-dominated and female engagement in outdoor pursuits have been limited to gentler and supervised activities [29,72,73,74].

### 4.3. Impacts of Gendered Barriers to PA

Although the participants’ engagement in PA was dependent on their individual belief and identities, all the participants commented on the perceived gendered PA limitations. A shared experience was how some boys used verbal harassment to belittle and put down the physical and sporting abilities of the girls in their school. This harassment resulted in the participants and their classmates losing confidence and motivation to participate in physical activities while in the presence of boys (i.e., during school). As school was a common and consistent location for PA engagement (and one of the only consistent avenues for PA for the nature girls), this may be limiting girls’ ability to engage in any activity in their day. This phenomenon of adolescent girls experiencing gendered limitations to PA both in the form of exclusion by boys, and insufficient support for girls PA in school, has been well documented in the literature [28,30,34].

School PE classes are a consistent site of PA for many Nova Scotian youth; however, they are also a site for the production and (re)production of heteronormative gendered embodiment, re-enforcing sociocultural gender norms. Joy et al. [75] found that although Nova Scotian schools are increasing adoption of gender inclusivity concepts, the experiences of Nova Scotian youth are still that of heteronormativity, (re)producing gendered bias. This experience is not isolated to Nova Scotia, as (re)production of gender norms within school PE classes has been widely identified in Western countries [76,77,78].

While all the participants commented on either experiencing or witnessing gendered PA exclusion, there were no actions to confront it. Some (Lillian, Ginny) accepted this behavior as the status quo, while Janet dealt with the verbal harassment by ignoring the comments. Actively resisting these overt experiences of gendered exclusion as well as more subtle heteronormative gender bias during PE can be difficult. Participatory action research has led to successfully empowering adolescent girls to enact their agency in resisting gendered exclusion and harassment [34]. Such participatory research practices, and similar ‘girls as co-leader’ programming may be able to give more girls the tools and confidence to speak out when confronted with gendered exclusionary situations.

While participants did experience gendered barriers, they also found ways in which to engage in meaningful PA. Girls navigate PA within the sociocultural pressure of hegemonic femininity, which is often contrary to traditional masculine forms of PA [29,79]. While none of the participants felt that ‘being a girl’ had any impact on their choices around physical activities, their PA choices and language illustrated the dominant feminine discourse. The most structured PAs engaged in by the participants were strongly associated with femininity (dance) or accepted as gender-neutral (soccer) [80]. The subconscious decision to choose ‘feminine appropriate’ activities is socially driven, as these activities are portrayed as being appropriate for girls to engage in while still maintaining femininity [9,30,31]. This portrayal of femininity is highly relevant to adolescent girls, as adolescents are in a transitionary time during which young girls are moving away from ‘childish’ activities, start to navigate highly complex socially constructed gender roles [29,46,81], and are navigating how to exist authentically within the social space [82]. Participation in activities that are outside of the expected gender roles can lead girls to experience social stigma around their gender, sexuality, and experience of a sense of ‘othering’, placing them outside of the socially accepted ‘norm’ [7,82].

While the dominant social discourse of femininity was seen in many of the participants’ experiences, there were also instances of individual agency against gender norms. The participation in baseball, combat sports, and hunting were outside of the ‘feminine’ and were engaged in by Jenny, Janet, Lillian, and Sharon. Being a girl participating in male-dominated activities requires constant identity management, as negotiating the discourse around gender-appropriate activities can leave girls open to social stigma and stereotyping [83,84]. The construction of social connectedness, as well as having female mentors and role models, has been found to support girls’ retention in male-dominated PA [7,85]. This was evidenced in the participation of girls-only teams with female leaders (baseball and combat sports) and was one way participants resisted the gendered bias and found identity in these activities.

### 4.4. Support Recommendations

This exploration of rural girls’ PA engagement highlighted several areas where social initiatives could help promote increased PA engagement for adolescent girls. Girls are navigating a complex personal and social gendered landscape while attempting to construct their PA identities. While increased opportunities for physical activity are needed for these adolescent girls, they also need support and role modeling to combat hegemonic femininity and gendered stereotypes to increase both their confidence and desires to participate in various physical activities. Programs focused on building adolescent girls’ self-confidence and agency can provide them with the tools needed to combat these gendered barriers. Concurrently, physical activity opportunities that are tailored to girls and more welcoming to girls are needed. Within PE spaces specifically, there is a need to not only *include* girls into the activity spaces but also to design curricula that are *inclusive* of all genders and abilities. Acknowledging how production of gender occurs during PE can aid teachers in designing classes that limit the privilege of heteronormative masculine activities over other forms of PA [45]. Similarly, community programming needs to both acknowledge how activities might re-enforce gendered stereotypes and address the creation of inclusive opportunities. Existing teams operating as co-ed can ensure meaningful supports are in place to ensure retention of adolescent girls. The implementation of female mentors and role models into the existing athletic programming, as well as increased gender training for youth coaches and PA leaders, can help to diminish negative PA experiences for adolescent girls [7,85]. To support girls who chose not to participate in co-ed teams, the construction of more girls-only teams would be better aligned to draw in girls who are wavering in their athletic identities due to perceived gendered barriers.

In addition to creating more and better PA opportunities and experiences, there must concurrently be increased parental support for PA, and rural-specific initiatives. The greatest reported barrier was transportation, which may be a significant hurdle for many rural families to overcome. The complex intersectionality that exists surrounding access to facilities, transportation requirements, and rural living cannot always be overcome by an individualistic approach, and therefore more social, structural support for increased affordable rural transportation will help rural parents support their children in their PA endeavors. Finally, not all girls want to participate in sport or other structured PA experiences. Rural-specific initiatives to increase unstructured PA in girls such as outdoor/nature-focused activities that do not rely on significant built environments (facilities, sidewalks, etc.) can help engage girls who either choose not to or cannot participate in more structured activities.

### 4.5. Strengths and Limitations

Exploring PA experience using a qualitative methodology, Heideggerian hermeneutic phenomenology, allowed for a deeper understanding of the lived experience, by providing not simply a measure of girls’ engagement in PA but allowing their individual experiences and voices to expand a more nuanced picture of the PA landscape in rural Nova Scotia for adolescent girls. This qualitative approach adds depth to the vast quantitative methodology that has been thus far undertaken in public health research and can therefore aid in the creation of more rural-specific supports and opportunities for PA engagement.

While this study provided important contributions to the PA literature, there are some limitations worth noting. First, all participants interviewed were Caucasian females, ages 10–13 years old, from middle-class homes. Therefore, the results found from this study are not necessarily transferable to adolescents from different racial or ethnic groups or ages. Additionally, due to the nature of phenomenology, the analysis is specific to the local, cultural, and historical context. Outcomes from this study cannot therefore be transferred to other locations, cultures, or times.

## 5. Conclusions

This study explored the lived experience of PA in rural Nova Scotian adolescent girls. The main findings centered around how rural living and parental support impacted the PA identity formation of the participants, and thus also impacted the level and modality of PA engagement. Linking all the themes was also the impact of gendered sociocultural expectations and hegemonic femininity on girls’ PA identity formation, PA levels, and choices. Adolescence is a time of significant change, both biologically and socially, and a time when there is a significant decline in girls’ PA engagement. Rural girls must navigate personal desires, sociocultural gender roles, peer influence, and parental support when engaging (or not) in PA, and therefore increased social and parental support is needed to ensure girls are able to engage in both the level and modality of PA of their choice.

## Data Availability

Data are unavailable due to privacy regulations surrounding interview transcripts.

## Abbreviations

The following abbreviations are used in this manuscript:

PA	Physical activity
HHP	Heideggerian hermeneutic phenomenology
MDPI	Multidisciplinary Digital Publishing Institute
DOAJ	Directory of Open Access Journals
TLA	Three-letter acronym
LD	Linear dichroism

## Appendix A

**Table A1:** Pseudonyms and ages of participants.

Pseudonyms	Age
Sharon	11
Janet	12
Jenny	11
Lillian	13
Sally	13
Ginny	12

**Table A2:** Example interview questions.

The term ‘physical activity’ may mean different things to different people; when you hear this term, what sorts of activities do you associate with physical activity?
Is doing physical activities important to you? Why/Why not?
What things get in the way of doing physical activities, like why do you not want to do some activities anymore, or if you do want to do some, why can you not? Can you provide an example of how this makes it difficult to do physical activity?
What helps you do the things you want to do? Can you provide an example of how this helps you do physical activity?
Do you feel like living in the county affects the activities that you do (compared to maybe living in a city or another part of NS)?

**Table A3:** Example reflexive questions used during analysis.

Author	Example Reflexive Questions
Valandra 2012 [60]	What have I done to ensure the interpretation I have presented in my writing is how my participants view the truth?
	How do my identities connect with the research? How do they shape the way I understand my interpretations of the experiences of my participants? What is my insider/outsider status?
	What are my implicit biases and attitudes shaped not only by ideology, but other layers of context/situatedness in which I find myself?
Duffy et al. 2021 [55]	How have my personal and professional experiences shaped what I know?
	How does my worldview influence the way I experience and/or construct this topic/idea?
	How do my social demographics shape my interpretation of the data collected?
	How can I privilege participants’ voices in the construction of new knowledge?

**Table A4:** Examples of raw data analysis into final themes.

Stage 1: Immersion (Reading and Note Taking on Individual Transcripts)	Stage 2: Understanding (Participant Level Codes)	Stage 3: Abstraction (Grouping Participant Codes into Sub-themes)	Stage 4: Theme Development	Stage 5: Illumination
“Well, we live pretty far away from a lot of stuff, and right now our town pool is closed. So if we wanna go swimming, we have to go all the way to New Glasgow. And the town skates are also really short, so if we want a longer skate, we have to go farther away.”	We live pretty far away	Lack of access to PA places	Rural life creates both supports and barriers to PA	It’s really nice to have space…but there a lot less options out here
“I think of like going outside and playing in the snow or in the grass, or playing with our dogs, maybe playing with some games in with my mom.”	Going outside and playing	Being outside IS PA for some girls	PA identity impacts what PA looks like to each girl	What PA looks like depends on your definition
“I liked some of them [PE modules] but some of them were a little intense just because I have a lot of sporty guys in my class and they kind of take over the entire gym and then it’s kind of awkward for a lot of the girls.”	They kind of take over the gym	Experience of PE is that some boys dominate and are gatekeeping athletics from the girls	Perceived gendered barriers can limit girls PA engagement	What do you do when the boys take over the gym
